# Factors associated with injuries among tornado victims in Yancheng and Chifeng, China

**DOI:** 10.1186/s12889-019-7887-6

**Published:** 2019-11-25

**Authors:** Qiangyu Deng, Yipeng Lv, Fangjie Zhao, Wenya Yu, Junqiang Dong, Lulu Zhang

**Affiliations:** 1 Office of Military Health Management, 941th Hospital of the Joint Logistics Support Force of the PLA, Xining, Qinghai Province China; 2 Office of Military Health Management, 909th Hospital of the Joint Logistics Support Force of the PLA, Zhangzhou, Fujian Province China; 3 Department of Military Health Management, College of Health Service, Second Military Medical University, 800 Xiangyin Rd, Shanghai, 200433 China

**Keywords:** Tornado, Trauma, Influencing factors, Empirical investigation

## Abstract

**Background:**

As extremely violent meteorological disasters, tornadoes often cause serious casualties. The study aims to analyze the characteristics and causes of tornado injuries in China under certain humanistic and geographical conditions.

**Methods:**

A random sampling questionnaire survey was developed and distributed to tornado victims from two separate occurrences: an Enhanced Fujita 4 tornado in Yancheng, and a Fujita 3 tornado in Chifeng. The information of demographic characteristics, disaster environment, and individual behaviors in victims was collected. Chi-square test and binary logistic regression were used to analyze influencing factor of injuries.

**Results:**

A total of 94 valid questionnaires (participation rate 95.9%) were finally collected in Yancheng tornado and 67 valid questionnaires (participation rate 93.1%) in Chifeng tornado. Residents’ annual income (OR = 0.10, 95% CI 0.02–0.50, *P* = 0.005), degree of house collapse (OR = 183.12, 95% CI 8.04–4173.34, *P* = 0.001) have a significant impact on the probability of injury. Differences in tornado disaster drill awareness (*P* = 0.009), individual behaviors (*P* = 0.011) and fear level (*P* = 0.011) significantly affected the incidence of trauma. Whether victims were indoors or not has no statistical difference on injuries in China.

**Conclusions:**

Our study clarifies risk factors and is conducive to the expansion of the investigation in tornado casualties. The government should improve the wind-resistance of residential buildings. Victims should participate in disaster prevention drills to take effective disaster avoidance actions.

## Background

Tornadoes are amongst the deadliest natural disasters and pose a major threat to worldwide public health. Between 1950 and 1994, over 4115 deaths and 70,063 injuries were reported as directly related to tornadic storms in the United States, with over 20 trillion US dollars’ worth of tornado-related damage [1].

**Fig. 1:**
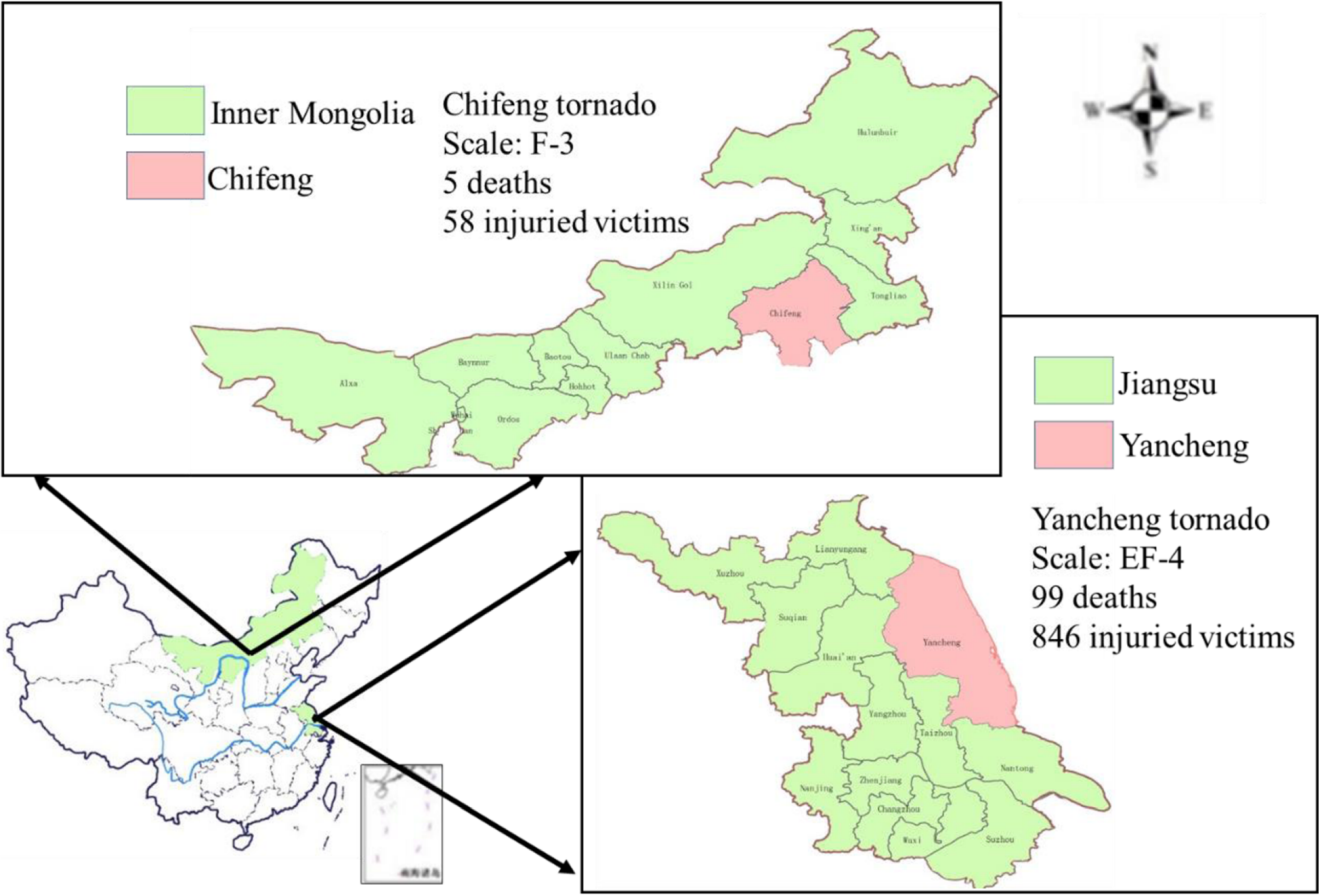
Location of the Yancheng Tornado in Jiangsu Province and the Chifeng Tornado in Inner Mongolia, China

Recent years, two violent tornadoes struck China, which caused massive loss of life and economic damage (Fig. 1). In the afternoon on June 23, 2016, an Enhanced Fujita 4 (EF-4) tornado tore through Yancheng City, Jiangsu Province, east China, with highest speed of 266 km/h (Enhanced Fujita scale categories for tornadoes range from 0 to 5, with the strongest being 5) [2, 3] (Fig. 1). The tornado affected 7 townships and 22 villages, resulting in 99 deaths and 846 wounded. In total, 29,371 houses, 2 primary schools, and 8 factories were damaged; more than 50,000 people were directly affected by the tornado [4]. This tornado was one of the largest disasters in recent decades in Jiangsu Province, and the deadliest tornado in China in nearly half a century [2, 5]. In the afternoon on August 11, 2017, a Fujita 3 (F-3, which is similar to EF-3) [6] tornado hit Keshiketeng County and Wengniute County in Chifeng City, Inner Mongolia (Fig. 1). This tornado affected 4 villages in 2 townships, causing 5 deaths and leaving 58 wounded. More than 30 houses collapsed, more than 2000 households were affected, and 1238 people were relocated [7].

At present, the research on the causes of the injuries and deaths caused by tornadoes mainly focuses on demographic factors, individual behaviors, architectural factors, and early warning information [8, 9]. For example, some studies [10–12] argue that old people are more vulnerable to injury in tornadoes. Maybe it is because their body is weaker and their movement ability is worse. Female was thought to be more vulnerable to injury [10, 11]. There are few studies on disaster avoidance behaviors, and there are controversies about the impact of fleeing by motor vehicle on injuries [1, 13–15]. Having safe helmet or car seats was a protective measure for children. Research shows that the majority of casualties are caused by the collapse of buildings and blunt force trauma from heavy objects [8, 11, 14]. Building with windows [12], or made by wood [1], or mobile car-house [13] increased the injury risk [1, 12]. However, sturdy shelter decreased the injury risk [13, 14]. Staying outside increased the injury risk [10], and basement of a house was a protective measure for residents [13]. That receipt of tornado warnings can reduce casualties [12, 13, 16]. However, at present China’s research on these aspects, especially pertaining to the complicated factors associated with tornado casualties, is basically non-existent.

Tornadoes in China mostly happen in coastal areas such as the Yangtze River Delta and Pearl River Delta [17]. These areas are densely populated with a high level of economic development, and therefore have the potential for mass casualties and huge economic losses. In terms of human characteristics, building environment, disaster prevention capabilities, tornadoes happened in China contribute to varying casualty results. Therefore, from the perspective of casualty prevention, it is especially important to analyze the factors influencing tornado injuries in China and reduce the probability of injuries by improving tornado casualty prevention and reduction. This study intends to analyze the factors influencing injury occurrences during the Yancheng tornado on June 23, 2016 and the Chifeng tornado on August 11, 2017; the factors that are herein examined include demographic characteristics, disaster environment, and individual behaviors. The study will provide clarification of risk factors and expand the research on tornado casualties.

## Methods

The respondents include victims from two separate tornado disaster events in China. There are three reasons we chose Yancheng and Chifeng. First, the two cities vary geographically. Chifeng is located in the highland part of Inner Mongolia, whereas Yancheng is located in the plains. Second, the structure of buildings differs between the two cities. The buildings in Yancheng consist of brick and cement, while they are made of mud tiles in Chifeng. Third, Yancheng has a much higher population density than Chifeng. In addition, these were the only areas in China that had been struck by fatal tornadoes in the past three years. The Yancheng tornado affected seven separate towns, from which we randomly chose four towns. The Chifeng tornado affected four villages, fewer than in Yancheng; therefore, we administered the survey to residents from all four villages. Hence, this study conducted a comprehensive survey of these eight areas. Since family members living together shared similar experiences, it was deemed to be more efficient to investigate individuals from different families to gather a more holistic view of the tornado and the related injuries.

**Fig. 2:**
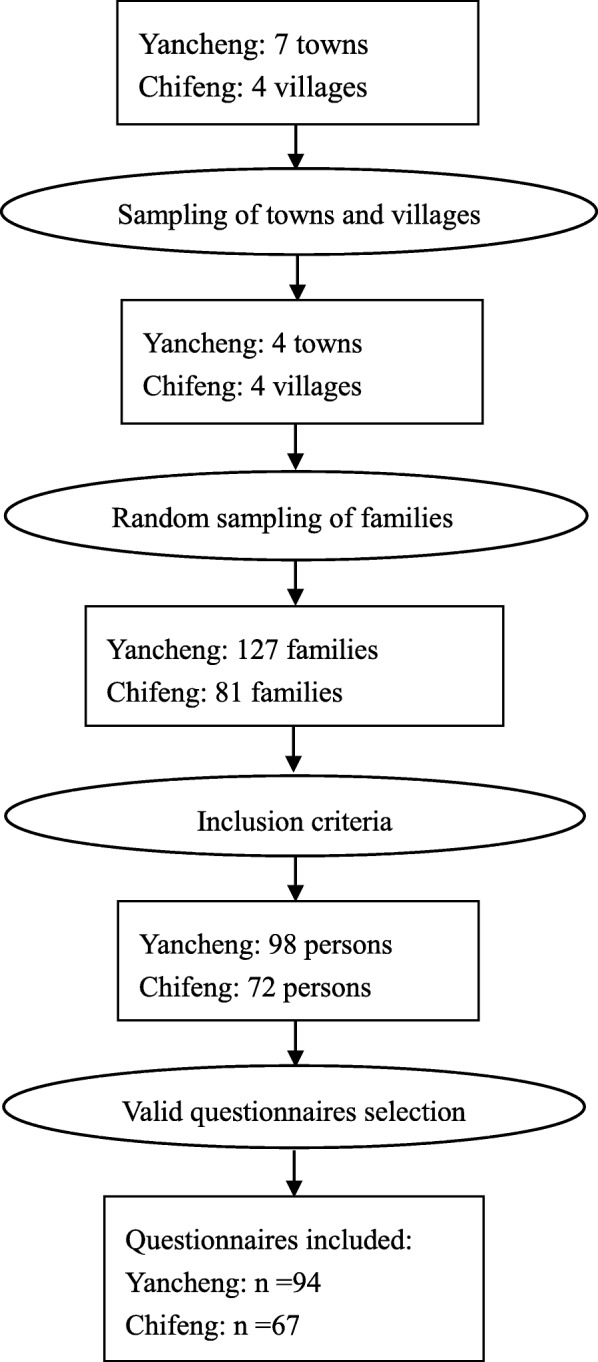
The flowchart about the sampling process

Figure 2 shows the flowchart about the sampling process. Two-step sampling was used in the research to avoid sampling bias. A random sampling method was used in both the first step (selecting the families) and the second step (selecting the specific family member). With the help of the Yancheng and Chifeng governments, random sampling was conducted through the household registration system in which every family was given a code [18]. Then, the family member whose birthday was closest to the investigation day was chosen as the participant. Parents completed the survey if the participants were under the age of 18. If the selected participant did not wish to participate for some reason, the individual with the second closest birthday was asked. This sampling method was used until we found a participant from each family. Inclusion criteria included experiencing tornado in Yancheng or Chifeng during the respective tornado disaster, and having their houses destroyed or damaged.

The questionnaire was designed by our research team. (Additional file 1) After modification by disaster emergency medical rescue experts and health service management experts, 10 victims were recruited for pre-testing (these 10 research results are not included in the final analysis). This was done before the formal survey administration in order to avoid a low response rate and unreasonable entry to ensure the quality of the questionnaire. The questionnaire has been widely used in disaster investigations, and has a good validity and reliability [19]. In this study, the Cronbach’s Alpha value is 0.796 for reliability, and the Kaiser-Meyer-Olkin value is 0.619 for validity. Based on other relevant studies that grouped factors of tornado injuries and deaths into demographic characteristics, circumstances, and behaviors [11, 13], the final questionnaire included three categories: first, the demographic characteristics of the victims, including sex, age, marital status, education level, and income level; second, the disaster environment, including being indoors (yes/no), living alone (yes/no), finding refuge (yes/no), the degree of housing collapse, house type, and years in which the house was built; and third, individual behaviors, including disaster avoidance behavior, fear level, and the need for tornado escape drills (yes/no).

The selected research method was a one-on-one questionnaire with the researcher filling out the questionnaire based on the victims’ oral responses. The investigators were master’s degree students in social medicine and public health service management. Unified training for investigators was provided in order to become familiar with the disaster situation and research considerations in advance. A local university volunteer was recruited as a language and culture guide. The survey was entirely anonymous. The interview time for each victim was 15–20 min. Four researchers from the research group traveled to the disaster-stricken area of Yancheng, Jiangsu Province, from July 12 to July 30, 2016, and to the disaster-stricken area in Chifeng, Inner Mongolia Autonomous Region, from August 15 to August 20, 2017.

### Statistical analysis methods

The questionnaires were uniformly coded one by one, and the data were double-entered by EpiData; inconsistent entries were proofread for clarification. Descriptive and statistical analyses were conducted to the questionnaire survey data for each of the two sets of tornado disaster victims. A uniform distribution Chi-square test was used to compare the differences in injuries among different groups of victims. For the two sets of samples, when 1 < theoretical frequency < 5, continuous correction was used, when the theoretical frequency < 1, the Fisher’s exact probability method was used for correction. Binary stepwise logistic regression analysis was used to analyze the influential factors of the victims’ injuries. The binary dependent variable is no injuries (0) and injury (1). The statistical software SPSS 21.0 (SPSS Inc., Chicago, IL, USA) was used, and the difference was considered statistically significant when *P* < 0.05.

## Results

In the Yancheng tornado, a total of 98 questionnaires were administered and 94 valid questionnaires were finally collected (participation rate 95.9%). In this case, 60 people were reported to have been injured in the tornado (63.8%). In the Chifeng tornado, a total of 72 questionnaires were administered, and 67 valid questionnaires (participation rate 93.1%) were collected; in this case, 29 people were reported to have been injured in the tornado (43.3%). The difference in injury rate between the two sets of victims of the tornado disasters is statistically significant (*P* = 0.010).

### Demographic characteristics

Table 1 shows that among the 94 victims in Yancheng, 46 were males (48.9%), 31 were over 65 (33.0%), and 73 were married (77.7%). There was a statistically significant difference in injuries among the victims from different annual income levels, and the probability of being injured with a high annual income is lower (*P* = 0.001).

**Table 1:** Impact of the victims’ demographic characteristics on their injuries

Category	Grouping	Yancheng			*P*	Chifeng			*P*
		Total number(%)	Number of uninjured(%)	Number of injured(%)		Total number(%)	Number of uninjured(%)	Number of injured(%)	
Sex	Male	46 (48.9)	20 (43.5)	26 (56.5)	0.149	53 (79.1)	30 (56.6)	23 (43.4)	0.971
	Female	48 (51.1)	14 (29.2)	34 (70.8)		14 (20.9)	8 (57.1)	6 (42.9)	
Age	< 20	3 (3.2)	2 (66.7)	1 (33.3)	0.336^a^	1 (1.5)	0 (0.0)	1 (100.0)	0.655 ^a^
	20–65	60 (63.8)	23 (38.3)	37 (61.7)		60 (89.6)	34 (56.7)	26 (43.3)	
	> 65	31 (33.0)	9 (29.0)	22 (71.0)		6 (9.0)	4 (66.7)	2 (33.3)	
Education level	Illiteracy	31 (33.0)	12 (38.7)	19 (61.3)	0.153	3 (4.5)	1 (33.3)	2 (66.7)	0.516 ^a^
	Primary school	32 (34.0)	8 (25.0)	24 (75.0)		10 (14.9)	4 (40.0)	6 (60.0)	
	Junior middle school	20 (21.3)	11 (55.0)	9 (45.0)		19 (28.4)	12 (63.2)	7 (36.8)	
	High school and above	11 (11.7)	3 (27.3)	8 (72.7)		35 (53.2)	21 (60.0)	14 (40.0)	
Annual income^c^	< 10,000	53 (56.4)	12 (22.6)	41 (77.4)	0.001 ^a^	53 (79.1)	30 (56.6)	23 (43.4)	1.000 ^a^
	10,000-50,000	37 (39.4)	18 (48.6)	19 (51.4)		12 (17.9)	7 (58.3)	5 (41.7)	
	> 50,000	4 (4.3)	4 (100.0)	0 (0.0)		2 (3.0)	1 (50.0)	1 (50.0)	
Marital status	married	73 (77.7)	24 (32.9)	49 (67.1)	0.215	59 (88.1)	34 (57.6)	25 (42.4)	0.977^b^
	Unmarried^d^	21 (22.3)	10 (47.6)	11 (52.4)		8 (11.9)	4 (50.0)	4 (50.0)	

^a^*Fisher’s exact probability test*

^b^*Yates’ continuity correction*

^c^*CҰ (Chinese Yuan)*

^d^*Including the divorced and widowed*

Compared to those surveyed in Yancheng, the proportion of people with high school and above education level was higher (53.2%), and the proportion of people with an annual income below 10,000 Chinese Yuan (79.1%) was higher, for the 67 victims in Chifeng. Demographic characteristics of the tornado victims in Chifeng were not significantly associated with the incidence of injuries.

### Disaster environment

Table 2 shows that among the victims from the Yancheng tornado, 14.9% were outdoors when the tornado occurred, and only 27.7% of the entire sample thought that they could find refuge. Those who experienced a complete collapse of their house accounted for 50.0%; residents living alone accounted for 21.3%. According to the data, victims who could not find refuge were more likely to be injured (*P* < 0.001). Additionally, it was found that the more serious the degree of housing collapse, the greater was the probability of the victims getting injured (*P* < 0.001).

**Table 2:** Impact of the disaster environment on victims’ injuries

Category	Grouping	Yancheng			*P*	Chifeng			*P*
		Total number(%)	Number of uninjured(%)	Number of injured(%)		Total number(%)	Number of uninjured(%)	Number of injured(%)	
Indoor	Yes	80 (85.1)	28 (35.0)	52 (65.0)	0.572	65 (97.0)	37 (56.9)	28 (43.1)	1.000^a^
	No	14 (14.9)	11 (42.9)	3 (57.1)		2 (3.0)	1 (50.0)	1 (50.0)	
Finding refuge	Yes	26 (27.7)	17 (65.4)	9 (34.6)	< 0.001	9 (13.4)	7 (77.8)	2 (22.2)	0.313^b^
	No	68 (72.3)	17 (25.0)	51 (75.0)		58 (86.6)	31 (53.4)	27 (46.6)	
The degree of housing collapse	Minor damage	4 (4.3)	4 (100.0)	0 (0.0)	< 0.001^a^	3 (4.5)	3 (100.0)	0 (0.0)	< 0.001^a^
	Moderate damage	12 (12.8)	7 (58.3)	2 (41.7)		23 (34.3)	18 (78.3)	5 (21.7)	
	Heavy damage	12 (12.8)	6 (50.0)	6 (50.0)		17 (25.4)	12 (70.6)	5 (29.4)	
	Partially collapsed	19 (20.2)	10 (52.6)	9 (47.4)		14 (20.9)	5 (35.7)	9 (64.3)	
	Completely collapsed	47 (50.0)	7 (14.9)	40 (85.1)		10 (14.9)	0 (0.0)	10 (100.0)	
House type	bungalow	75 (79.8)	25 (33.3)	50 (66.7)	0.255	67 (100.0)	38 (56.7)	29 (43.3)	——^c^
	build	19 (20.2)	16 (47.4)	3 (52.6)		0 (0.0)	0 (0.0)	0 (0.0)	
Years in which the house was built	70s and before	8 (8.5)	3 (37.5)	5 (62.5)	0.387	2 (3.0)	1 (50.0)	1 (50.0)	0.636 ^a^
	80s	44 (46.8)	12 (27.3)	32 (72.7)		17 (25.4)	11 (64.7)	6 (35.3)	
	90’s	27 (28.7)	12 (44.4)	15 (55.6)		26 (38.8)	16 (61.5)	10 (38.5)	
	After 2000	15 (16.0)	7 (46.7)	8 (53.3)		22 (32.8)	10 (45.5)	12 (54.5)	
Living alone	Yes	20 (21.3)	8 (40.0)	12 (60.0)	0.688	7 (10.5)	6 (85.7)	1 (14.3)	0.217^b^
	No	74 (78.7)	26 (35.1)	48 (64.9)		60 (89.6)	32 (53.3)	28 (46.7)	

^a^*Fisher’s exact probability test*

^b^*Yates’ continuity correction*

^c^*The sample is too small to calculate a valid P value*

Compared with the Yancheng victims, the proportion of outdoor residents in Chifeng was lower (3.0%). The proportion of those who thought that they could find refuge was lower (13.4%); also, the proportion of completely collapsed houses was lower (14.9%). All local house types are cottages, with a higher proportion of newly-built houses after 2000 (32.8%). Similar to the Yancheng residents, for the victims of the Chifeng tornado, the higher the degree of housing collapse, the greater the probability of victims getting injured (*P* < 0.001).

### Individual behavior

Table 3 shows that when the Yancheng tornado occurred, 55.3% of the victims chose to flee indoors and 20.2% of the victims chose to use their bodies to hold the doors and windows. Results indicated that 64.9% of the victims were extremely frightened when the disaster occurred, and the tornado event made 86.2% of the victims think it was necessary to conduct tornado escape drills. There is no statistical significance among different disaster avoidance behaviors, fear level, or the need for tornado escape drills.

**Table 3:** Impact of individual behavior on victims’ injuries

Category	Grouping	Yancheng			*P*	Chifeng			*P*
		Total number(%)	Number of uninjured(%)	Number of injured(%)		Total number(%)	Number of uninjured(%)	Number of injured(%)	
Disaster avoidance behavior	Stand still	19 (20.2)	8 (42.1)	11 (57.9)	0.838 ^a^	7 (10.5)	4 (57.1)	3 (42.9)	0.011^a^
	Fled to indoor	52 (55.3)	18 (34.6)	34 (65.4)		26 (38.8)	9 (34.6)	17 (64.4)	
	Hold doors and windows	19 (20.2)	6 (31.6)	13 (68.4)		33 (49.3)	24 (72.7)	9 (27.3)	
	Run away from the tornado	4 (4.3)	2 (50.0)	2 (50.0)		1 (1.5)	1 (100.0)	0 (0.0)	
Fear level	No fear	5 (5.3)	4 (80.0)	1 (20.0)	0.137	3 (4.5)	2 (66.7)	1 (33.3)	0.011^a^
	A little fear	4 (4.3)	2 (50.0)	20 (50.0)		2 (3.0)	2 (100.0)	0 (0.0)	
	General fear	7 (7.5)	2 (28.6)	5 (71.4)		3 (4.5)	3 (100.0)	0 (0.0)	
	Severe fear	17 (18.1)	8 (47.1)	9 (52.9)		7 (10.5)	5 (71.4)	2 (28.6)	
	Extreme fear	61 (64.9)	18 (29.5)	43 (70.5)		52 (77.6)	26 (50.0)	26 (50.0)	
The Need for tornado escape drills	Yes	81 (86.2)	31 (38.3)	50 (61.7)	0.455^b^	50 (74.6)	33 (66.0)	17 (34.0)	0.009
	No	13 (13.8)	3 (23.1)	10 (76.9)		17 (25.4)	5 (29.4)	12 (70.6)	

^a^*Fisher’s exact probability test*

^b^*Yates’ continuity correction*

Compared with the victims in Yancheng, the rate of victims choosing to use their bodies to hold doors and windows is higher (49.3%), and the rate of fleeing indoors is lower (38.8%) in Chifeng. The proportion of victims with extreme fear is even higher (77.6%), and the proportion of people who were made by the tornado thinking that it was necessary to conduct tornado escape drills is lower (74.6%). According to the statistical test, the disaster avoidance behaviors of residents significantly affected the occurrence of injury (*P* = 0.011), residents who fled indoors had a higher percentage of injuries, and residents who used their bodies to hold doors and windows had a lower percentage of injuries. The higher the fear level in the victims was, the greater the probability of injury (*P* = 0.011). Victims who considered it necessary to conduct tornado escape drills were less likely to be injured (*P* = 0.009).

### Factors affecting injury

In Table 4, logistic regression analysis was performed on the factors associated with the victims’ injuries, which include variables of demographic characteristics, disaster environment, and individual behaviors. Regression analysis showed that for the victims of the Yancheng tornado, family income level (OR = 0.10, 95% CI 0.02–0.50) and the degree of housing collapse (OR = 183.12, 95% CI 8.04–4173.34) had a significant impact on the victims’ injuries.

**Table 4:** Logistics regression analysis of influential factors on victims’ injuries in Yancheng tornado^a^

Influencing factors	OR (95% CI)	*p*
Gender (Ref: Male)	3.44 (0.73, 16.17)	0.118
Age (Ref: < 20)		
20–65	4.21 (0.02, 935.30)	0.602
> 65	4.41 (0.02, 1055.00)	0.596
Education level (Ref: Illiteracy)		
Primary school	0.78 (0.11, 5.36)	0.794
Junior middle school	0.42 (0.05, 3.60)	0.426
High school and above	1.11 (0.10, 12.24)	0.930
Income level (Ref: < 10,000)		
> 10,000	0.10 (0.02, 0.50)	0.005
Marital status (Ref: Unmarried)	2.33 (0.31, 17.56)	0.413
Living alone (Ref: No)	0.75 (0.09, 6.39)	0.794
Indoor (Ref: No)	0.33 (0.03, 3.11)	0.329
House type (Ref: Bungalow)	11.74 (1.35, 102.33)	0.026
House building years (Ref: 70s and before)		
80s	1.31 (0.08, 21.70)	0.850
90’s	0.23 (0.01, 4.39)	0.326
After 2000	2.59 (0.08, 85.49)	0.594
Fear level (Ref: No fear)		
A little fear	5.90 (0.02, 1839.14)	0.545
General fear	10.50 (0.10, 1106.22)	0.322
Severe fear	3.80 (0.04, 382.15)	0.571
Extreme fear	2.43 (0.03, 176.67)	0.684
disaster avoidance behavior (Ref: Stand still)		
Fled to indoor	1.74 (0.26, 11.56)	0.567
Hold doors and windows	0.84 (0.089, 7.89)	0.878
Run away from the tornado	0.05 (0.00, 240.16)	0.481
The degree of housing collapse (Ref: Minor damage)		
Moderate damage	10.50 (0.76, 144.56)	0.079
Partially collapsed	12.25 (0.94, 160.11)	0.056
Completely collapsed	183.12 (8.04, 4173.34)	0.001
Finding disaster prevention building (Ref: No)	0.246 (0.047, 1.274)	0.095
Need tornado escape drills (Ref: No)	0.098 (0.004, 2.325)	0.151

Note. CI = confidence interval; OR = Odds Ratio

*.*P* < 0.05

## Discussion

### Demographic characteristics affecting injuries

For the tornado victims in Yancheng, a higher income level was associated with lower odds of injury (OR = 0.10, 95% CI = 0.02, 0.50). Households with high income may have relatively good building resilience and personal disaster prevention equipment, and their ability to endure disasters is relatively strong [20]. Low income may reduce the likelihood of disaster victims receiving relevant early weather warning information due to a lack of Internet, and the ability to take effective disaster avoidance actions. It is suggested that the government should increase financial relief funds to help victims improve the wind resistance of houses, and improve personal tornado disaster prevention equipment, such as helmets and shelters. It is also necessary to increase investment in disaster prevention for high risk groups such as the poor, which can reduce injuries and the overall public health expense.

### Collapsed buildings are important factors for injuries

Considering the effects of tornadoes on public health, it is necessary to increase the wind resistance of houses within the community, such as by building basements and storm shelters.

In this study, whether victims were indoors or not has no statistical difference on injuries, but rather the degree of housing collapse was related to injuries, because the house in China were likely to collapse facing tornado. This is different from studies in the United States and other countries. In most American studies, being outdoors is a risk factor for injuries [10, 14]. There are several reasons that may be the case. Primarily, injuries are related to the severity of building collapse during tornado disasters in China. In the Yancheng tornado, 50.0% of the victims’ houses completely collapsed, and 14.9% also experienced this in Chifeng. The houses in the disaster-stricken areas have poor wind-resistant capabilities and seriously collapsed, indicating that houses are not reliable for protection from tornados. The degree of housing collapse (OR = 183.12, 95% CI = 8.04, 4173.34) was closely related to the victims’ injuries in Yancheng tornado. This supports the conclusions made in the investigation on the factors associated with tornado injuries in Bangladesh in 2005 [10].

The degree of housing collapse is related to various factors such as wind level, house structure, and the age of the house. This suggests that in preparation for the prevention and mitigation of tornadoes, wind resistance should be the focus. In addition, Bohonos [1] concluded that in the United States, high-rise buildings are more dangerous to victims in tornadoes than low-rise buildings. This explains the higher injury ratio in Yancheng than in Chifeng; the collapse of buildings in Yancheng was more serious, and there were no high-rise buildings in Chifeng.

These conclusions can also be applied to other building features in China. The wind resistance of windows in the buildings in rural China is weaker, and this allows greater opportunity for debris to harm occupants hiding indoors. During the tornadoes, many indoor victims used their bodies to hold doors and windows, increasing the potential for injury during a high-intensity tornado. Schmidlin [12] and Niederkrotenthaler [13] both found that rooms with windows are a risk factor for injury (*P* < 0.1).

In addition, studies in the United States reveal that the basement is a protective factor for tornadoes (OR = 0.13; 95% CI: 0.04–0.40) [13, 21]. One reason for the higher death rate during the Joplin tornadoes in Joplin, Missouri, in 2011 is that the ratio of local houses that have basements is low [22]. Similarly, almost no basements exist in the Yancheng and Chifeng tornado areas, which reduced the houses’ protection function; this is a primary reason why residents both inside and outside of the house have a similar likelihood of being injured. In some parts of the United States, shelters have been specifically built for storms, and studies have shown adequate storm shelters to be important to residents of tornado-prone areas, including the provision of financial assistance for building underground shelters or above ground safe rooms [23].

### Disaster prevention can reduce injuries

In the case of the Chifeng tornado, residents who believed that disaster escape drills are necessary were significantly less likely to incur injuries (*P* = 0.009). This indicates that residents who think disaster escape drills are necessary have a higher awareness of disaster prevention and reduction techniques. They tend to pay more attention to disaster-related information and scientific knowledge, so as to take more active and effective measures to prevent disasters and reduce their probability of being injured. In China, the occurrence of tornadoes is infrequent, and most residents do not have any experience with tornado escape drills. It is suggested that disaster escape drills should be appropriately conducted for those living in high-risk areas to enhance the awareness of disaster prevention.

Personal awareness of disaster prevention can affect behavior and fear levels. The proportion of casualties fleeing indoors is significantly higher in the Chifeng tornado (*P* = 0.011), which is related to the house structure in Inner Mongolia. The wall tiles and roof tiles in this area are mud-bonded; thus, their resistance to tornadoes is weak and they do not provide sufficient protection for indoor occupants. There was a low proportion of people injured who were using their body against doors and windows (*P* = 0.011), which is opposite to the results from the Yancheng EF-4 tornado. This suggests that in less intense tornado disasters (F-3), holding windows and doors closed is an effective technique in preventing injury. However, in the case of a tornado disaster with greater intensity, such behavior is not a preventative technique and should be avoided especially since you do not know the strength of the tornado. The higher the victim’s fear level, the greater the probability of injury (*P* = 0.011). Psychological fear often increases the risk of injury [19].

### Limitations

There remain some limitations of this study. First, the recall bias still existed, which came from interviewing survivors (as worse hit person might have died from injuries sustained). Second, the sample size of Chifeng tornado is small, so we cannot do logistic regression analysis for the Chifeng tornado. In subsequent studies, we will pay more attention to worse hit persons and the death, and the sample size for reducing recall bias and increasing reliability of analysis respectively.

## Conclusions

Tornadoes are amongst the deadliest natural disasters and pose a major threat to worldwide public health. This is the first study to investigate factors associated with tornado injuries in China, which is an important topic to consider for the reduction of casualties. Our findings have some important implications for public health. First, governmental disaster preparedness is very important for community public health in disaster areas. It is necessary to provide reinforcements for the prevention of tornado injuries, especially for high risk groups such as the poor and the elderly. One way to do this would be to improve community shelters and build tornado-safe shelters and basements according to standardized safe building criteria. Second, public health preparedness education should be implemented. The use of disaster prevention programs should be increased, which can help victims to learn coping behaviors while also reducing their fears. Personal and family preparedness planning should be employed, and access to warnings and safe shelters should be enhanced. Third, warning preparedness and hospital plans are critical for community safety. Community tornado warning alarms should be improved, and hospital emergency disaster rescue plans for tornadoes should be standardized to improve rescue effectiveness and efficiency.

## Supplementary information

**Additional file 1.** The questionnaire of tornado victims.

## Data Availability

No additional data are available.

## Abbreviations

EF-4: Enhanced Fujita 4
F-3, similar to EF-3: Fujita 3
