# Supplementary material for: Factors associated with injuries among tornado victims in Yancheng and Chifeng, China
Source: BMC Public Health. 2019 Nov 25;19:1556. doi: 10.1186/s12889-019-7887-6 (PMC6878644; doi:10.1186/s12889-019-7887-6)
Supplement: Supplementary file 1 — Additional file 1. The questionnaire of tornado victims. [file 12889_2019_7887_MOESM1_ESM.doc]

**The questionnaire of tornado victims**

Dear sir / madam

Hello! Thank you for participating in our survey! This survey uses questionnaire to know about the disaster situation of victims and the responsiveness and accessibility of medical rescue after tornado, which provides an important basis for national tornado ‘disaster prevention, mitigation and relief’. The content of the survey is only for scientific research. We hope you answer all the following questions truthfully, objectively and completely. Thank you for your cooperation again!

**Demographic characteristics**

1. Sex: □Male □Female

2. Age: years old

3. Education level: □Illiteracy □Primary school

£Junior middle school □High school and above

1. Annual income(C￥ Chinese Yuan)

□< 10,000 £10,000-50,000 □> 50,000

1. Marital status: £Married £Unmarried

**Disaster environment**

1. When the tornado happened, were you in Indoor?

£Yes £No

1. When the tornado happened, could you find refuge?

£Yes £No

1. The degree of your house collapse:

£Minor damage

£Moderate damage

£Heavy damage

£Partially collapsed

£Completely collapsed


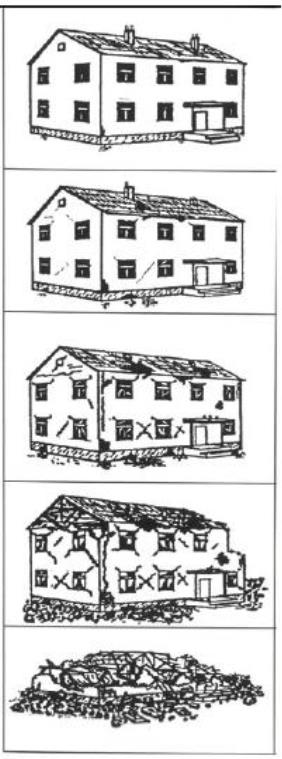


1. Your house type: £Bungalow □ Build
2. Years in which the house was built:

£70s and before £80s £90's £After 2000

6. Are you live alone?

£Yes £No

**Individual behavior**

1. When the tornado happened, your disaster avoidance behavior:

£Stand still £Fled to indoor

£Hold doors and windows £Run away from the tornado

1. When the tornado happened, your fear level:

£No fear £A little fear £General fear

£Severe fear £Extreme fear

3. Do you need tornado escape drills?

£Yes £No
